# Perceptions on the management of varices and on the use of albumin in patients with cirrhosis among GI specialists in Austria

**DOI:** 10.1007/s00508-020-01769-9

**Published:** 2020-12-03

**Authors:** Nikolaus Pfisterer, Caroline Schmidbauer, Florian Riedl, Andreas Maieron, Vanessa Stadlbauer, Barbara Hennlich, Remy Schwarzer, Andreas Puespoek, Theresa Bucsics, Maria Effenberger, Simona Bota, Michael Gschwantler, Markus Peck-Radosavljevic, Mattias Mandorfer, Christian Madl, Michael Trauner, Thomas Reiberger

**Affiliations:** 1 Division of Gastroenterology and Hepatology, Department of Internal Medicine III, Vienna General Hospital and Medical University of Vienna, Währinger Gürtel 18–20, 1090 Vienna, Austria; 2 Vienna Hepatic Hemodynamic Lab, Medical University of Vienna, Vienna, Austria; 3 4. Medizinische Abteilung für Gastroenterologie und Hepatologie, Klinik Landstraße / Krankenanstalt Rudolfstiftung, Wien, Austria; 4 4. Medizinische Abteilung für Gastroenterologie und Hepatologie, Klinik Ottakring / Wilhelminenspital, Wien, Austria; 5 Fakultät für Medizin, Sigmund Freud Universität Wien, Wien, Austria; 6 Klinische Abteilung für Innere Medizin 2, Universitätsklinikum St. Pölten, St. Pölten, Austria; 7 Klinische Abteilung für Gastroenterologie und Hepatologie, LKH-Univ. Klinikum Graz, Graz, Austria; 8 Innere Medizin und Gastroenterologie (IMuG) mit zentraler Aufnahme und Erstversorgung (ZAE), Klinikum Klagenfurt am Wörthersee, Klagenfurt am Wörthersee, Austria; 9 Abteilung für Innere Medizin II, Krankenhaus der Barmherzigen Brüder, Eisenstadt, Austria; 10 Abteilung für Innere Medizin I, Universitätsklinikum Innsbruck, Innsbruck, Austria; 11 Christian-Doppler Laboratory for Portal Hypertension and Liver Fibrosis, Medical University of Vienna, Vienna, Austria

**Keywords:** Survey, Practice, Albumin, Non-selective beta blockers, Transjugular intrahepatic portosystemic shunt

## Abstract

**Background:**

Portal hypertension (PH) causes severe complications in patients with liver cirrhosis, such as variceal bleeding and ascites; however, data on the knowledge and perceptions on guideline recommendations for the management of varices and the use of albumin is scarce.

**Methods:**

We designed two structured surveys on (i) the management of varices and (ii) the use of albumin for Austrian physicians of specialized Gastro-Intestinal (GI) centers. The interviewed physicians were confronted spontaneously and provided *ad hoc* responses to the questionnaire.

**Results:**

In total, 158 surveys were completed. Interestingly, many specialists (30%) would recommend a follow-up gastroscopy after 1 year in patients with compensated cirrhosis without varices (i.e., overtreatment). For small varices, 81.5% would use non-selective beta blockers (NSBB) for primary prophylaxis (PP). For PP in patients with large varices, endoscopic band ligation (EBL) plus NSBB was preferred by 51.4% (i.e., overtreatment). Knowledge on the indication criteria for early TIPS (transjugular intrahepatic portosystemic shunt) was reported by 54.3%, but only 20% could report these criteria correctly. The majority (87.1%) correctly indicated a preference to use NSBB and EBL for secondary prophylaxis (SP).

The majority of participating gastroenterologists reported no restrictions on the use of albumin (89.8%) in their hospitals. Of the interviewed specialists, 63.6% would use albumin in patients with SBP; however, only 11.4% would use the doses recommended by guidelines. The majority of specialists indicated using albumin at the recommended doses for hepatorenal syndrome (HRS-AKI, 86.4%) and for large volume paracentesis (LVP, 73.3%). The individual responses regarding albumin use for infections/sepsis, hyponatremia, renal impairment, and encephalopathy were heterogeneous.

**Conclusion:**

The reported management of PH and varices is mostly adherent to guidelines, but endoscopic surveillance in patients without varices is too intense and EBL is overused in the setting of PP. Knowledge on the correct use of early TIPS must be improved among Austrian specialists. Albumin use is widely unrestricted in Austria; however, albumin is often underdosed in established indications.

**Electronic supplementary material:**

The online version of this article (10.1007/s00508-020-01769-9) contains supplementary material, which is available to authorized users.

## Introduction

Portal hypertension (PH) causes severe complications in patients with cirrhosis, including ascites, acute variceal bleeding (AVB), hepatorenal syndrome (HRS-AKI), and spontaneous bacterial peritonitis (SBP) [1–3]. Thus, management of PH requires a systematic approach as well as expert knowledge on the prevention and treatment of PH-associated complications in order to improve patient outcome and quality of life. Adherence to guidelines was associated with improved outcomes [4, 5]. The European Baveno VI guidelines and the Austrian Billroth III consensus provide detailed recommendations for the management of PH to guide physicians in their daily clinical practice [1, 2]. Moreover, the European Association for the Study of the Liver (EASL) issued clinical practice guidelines on the management of ascites and its complications and more recently, decompensated liver cirrhosis [3, 6].

Around 30% of patients with cirrhosis develop esophageal varices and despite improvements in the management of variceal hemorrhage, bleeding-related mortality remains as high as 15–20% [7–11]. Importantly, the rate of rebleeding is up to 60% if no adequate secondary prophylaxis is provided [12]. For primary prophylaxis of variceal bleeding, current international guidelines recommend either nonselective beta blockers (NSBB) or endoscopic band ligation (EBL); however, Austrian guidelines indicate a preference for NSBB [2, 13]. Secondary prophylaxis of variceal rebleeding should be performed by combination treatment (NSBB plus EBL) [2, 13]; however, a retrospective study in Austria showed that up to one third of patients received secondary prophylaxis with EBL alone [14]. Among these, medical conditions representing contraindications to NSBB could explain the lack of NSBB therapy in only 25.8% of cases [14, 15]. Furthermore, more than half of the patients in this bicentric study received EBL plus NSBB in primary prophylaxis, although the current guidelines do not recommend this regimen in this setting [2, 13, 14]. It remained largely unknown why adherence to the guidelines was so low. In addition, recent data indicates underutilization of early transjugular intrahepatic portosystemic shunt (TIPS) in clinical routine, although its benefits are well established [16, 17]. Importantly, there is an ongoing controversy regarding the use of NSBB in patients with refractory ascites with or without spontaneous bacterial peritonitis (SBP) [18–21], which likely impacts on the use of NSBB for bleeding prophylaxis by treating physicians, especially in patients with a history of ascites.

The use of albumin is recommended in several indications related to complications of PH [1, 2]. The EASL clinical practice guidelines and Billroth III consensus guidelines recommend the use of albumin in patients with cirrhosis undergoing a large-volume paracentesis (LVP) to prevent paracentesis-induced circulatory dysfunction (PICD), as well as in patients with SBP or hepatorenal syndrome (HRS-AKI) [1, 2, 22, 23]. In addition to the beneficial effects of albumin on renal perfusion, long-term administration of albumin may improve outcomes in patients with ascites [24, 25]. The use of albumin was reported to reduce systemic inflammation, improve hemodynamics and ameliorate neurological symptoms of hepatic encephalopathy (HE) [26–29]. Furthermore, albumin is also used in critically ill patients for circulatory support and has a well-established safety profile [30]; however, the latter indications in patients with cirrhosis are controversially discussed among experts.

Therefore, we aimed to capture the perceptions regarding the management of PH and the use of albumin among physicians caring for patients with cirrhosis in Austria in comparison with current Billroth III recommendations [2]. The surveys covered both knowledge and adherence to guidelines as well as questions regarding controversial issues.

## Methods

We designed two structured surveys which were handed out to physicians of specialized GI centers in Austria, who regularly treat patients with cirrhosis.

The physicians were spontaneously interviewed at various national meetings between June 2018 and November 2019. It was assured that they could not check the guidelines before answering the questions.

The first survey (*survey‑A*, *see* “Supplementary Material”), assessed important aspects regarding prophylaxis and treatment of patients with esophageal varices (EV) in their daily clinical practice.

*Survey‑A* consisted of 10 questions about the preferred strategy for screening for varices, primary prophylaxis (PP) of EV, surveillance after EBL, treatment of AVB including early TIPS, and secondary prophylaxis (SP) of EV. The questionnaire included case examples with response options to assess how the physician would use nonselective beta blockers (NSBB) in patients with cirrhosis and portal hypertension.

A second survey (*survey‑B*, *see* “Supplementary Material”) addressed important aspects regarding access/reimbursement and the usage and dosage of albumin in their daily clinical practice. *Survey‑B* consisted of 9 questions including 4 cases about albumin: indications, dosages and access to albumin at their hospital. *Survey‑B* also included case examples with response options regarding the use of albumin in patients with cirrhosis and paracentesis, AVB, hyponatremia, and SBP.

Responses to questions that did not address controversial issues were categorized in correct, wrong or borderline. Answers close to the correct recommendation (i.e., minor deviations in dosage) according to the current guidelines were classified as borderline [1, 2]. If a question was not answered from a physician, the question was marked as not available (n/a).

Information about the population and urban-rural typology of Austria was collected from www.statistik.at (Statistics Austria) [31, 32].

### Statistic

Only descriptive statistics were used and all statistical analyses and illustrations were computed using GraphPad Prism 6 (GraphPad Software, La Jolla, CA, USA). The number of respective given answers were expressed as absolute numbers and/or percentages.

### Ethics

No patients were involved in this survey, and thus, ethics committee approval was not required.

## Results

### Demographic characteristics (Fig. 1 and 2, Table S2 in Supplementary Material)

**Fig. 1:**
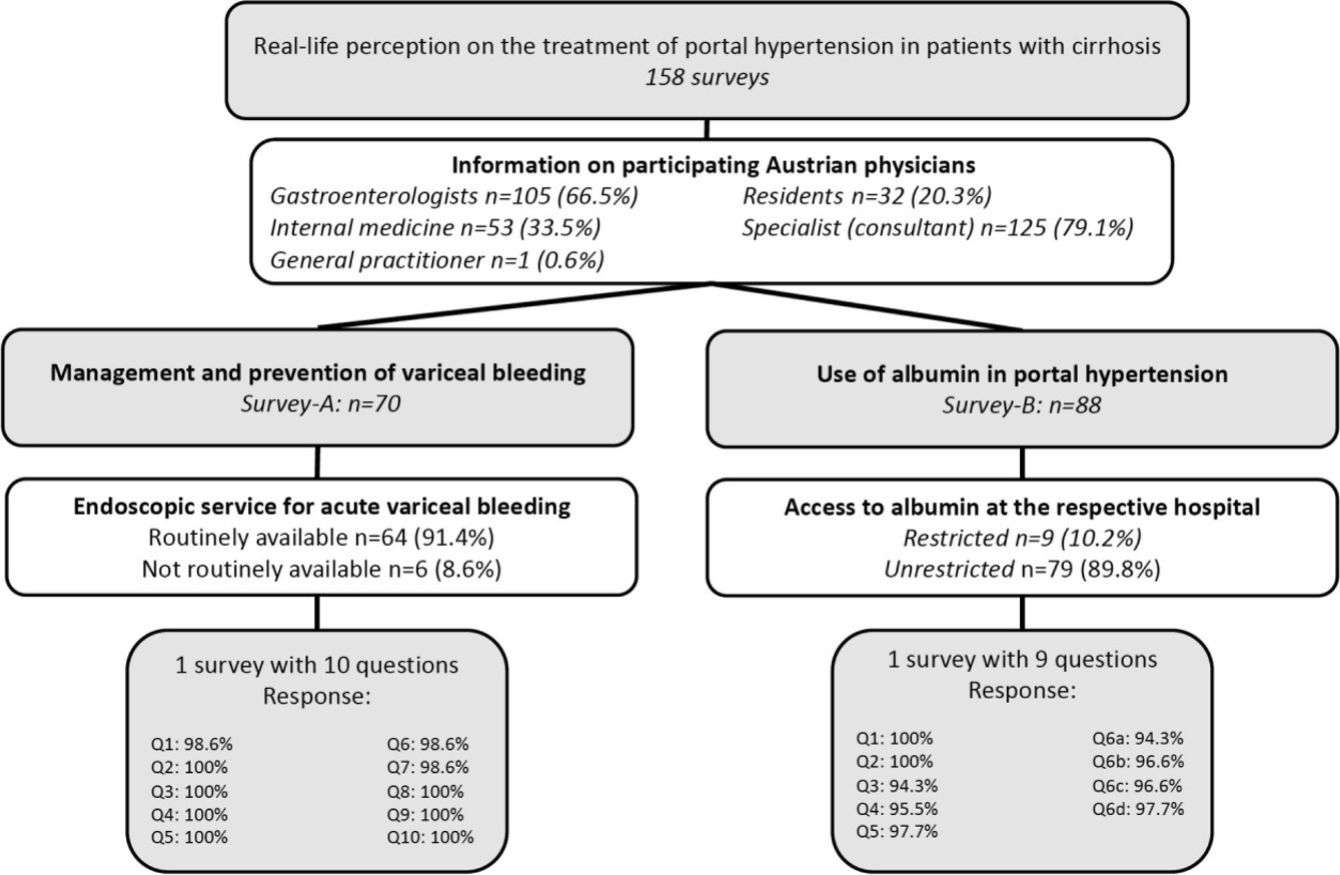
Flow chart depicting information on participating doctors. *Q* Question

**Fig. 2:**
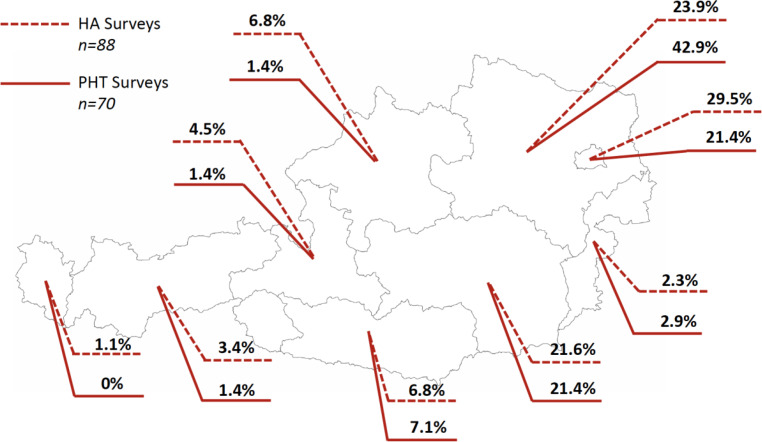
Geographical distribution of returned questionnaires across Austria. *n* number of questionnaires, *HA* survey about the use of albumin in patients with portal hypertension, *PHT* survey about the management and prevention of variceal bleeding and other complication of portal hypertension. Source of the map of Austria: https://d-maps.com/m/europa/austria/autriche_de/autriche_de46.pdf

In total, 70 physicians completed the survey on management of varices (*survey‑A*) and 88 physicians completed the survey (*survey‑B*) on the use of albumin in Austrian hospitals. Among 158 surveys, 126 (79.7%) were answered by specialists (consultants) while 32 (20.3%) were residents/fellows. Importantly, 105 (66.5%) participants were specialized in gastroenterology and hepatology, while 53 (33.5%) of participants were in training/specialized in internal medicine.

Overall, 40 (25.3%) physicians only reported the state (e.g. Burgenland, Carinthia, etc. …) of their workplace. Among the remaining physicians, 91 (57.6%) stated to work in state capitals (e.g. Wien, Linz) and 27 (17.9%) physicians worked in smaller cities (e.g. Melk).

Of the 70 interviewed physicians (*survey‑A*) 6 (8.6%) reported not having access to endoscopic services for the treatment of AVB, whereas 79 of 88 (89.8%) interviewed physicians (*survey‑B*, question 1) reported having unrestricted access to albumin. Only 4 physicians (4.5%) provided a more detailed explanation for restricted use of albumin: Two physicians had to fill in specific request forms to obtain albumin, in one institution albumin is only used in intermediate/intensive care, and one physician reported to have no possibility to use albumin in private practice.

**Fig. 3:**
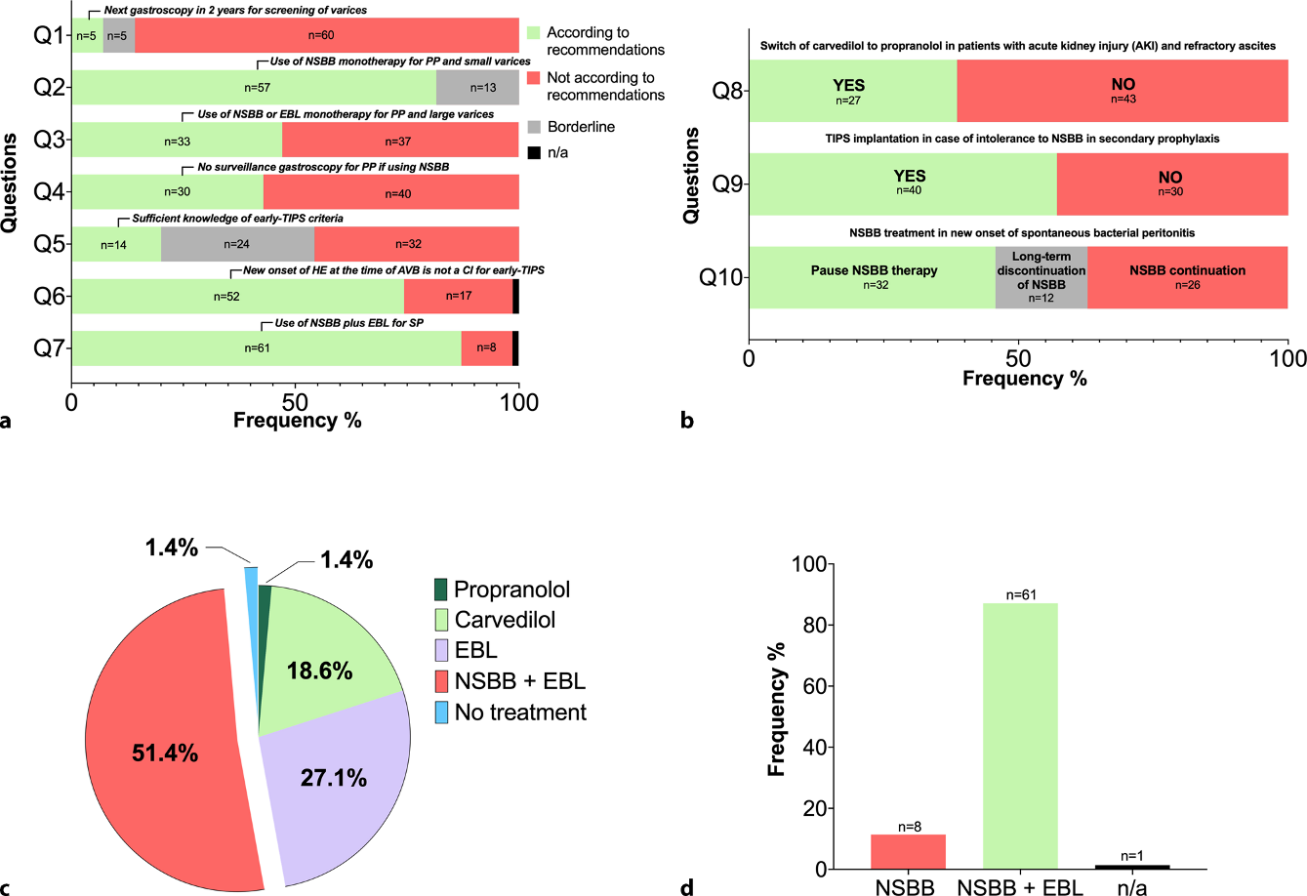
Responses given to questions regarding the management of varices and variceal bleeding in *survey‑A* to questions with **a** strong recommendations by guidelines and **b** weak recommendations by guidelines. **c** Responses on the preferred choice for primary bleeding prophylaxis in patients with compensated liver cirrhosis (Child-Pugh Class A6) with large varices and red spot signs. **d** Responses on the preferred choice for secondary prophylaxis of variceal bleeding. *Q* question, *n/a* not answered, *n* number of surveys, *NSBB* nonselective beta blockers, *TIPS* transjugular intrahepatic portosystemic shunt, *EBL* endoscopic band ligation, *AVB* acute variceal bleeding, *PP* primary prophylaxis, *SP* secondary prophylaxis, *CI* contraindication

**Fig. 4:**
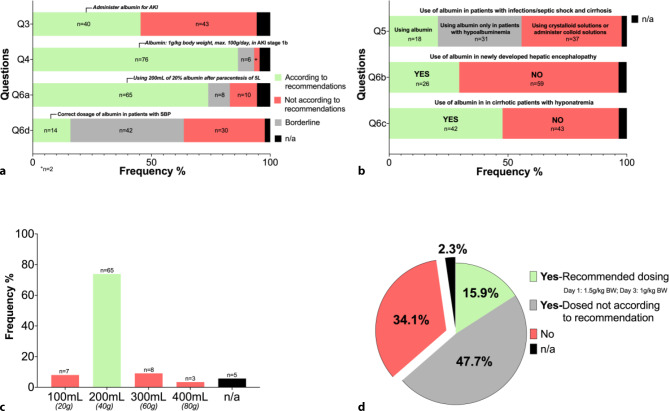
Responses of *survey‑B* on albumin use in patients with liver cirrhosis with **a** strong recommendations and **b** weak recommendations/controversial recommendations. Responses on use of albumin in **c** patients with paracentesis of a volume of 5L ascitic fluid and in **d** patients with spontaneous bacterial peritonitis. *Q* question, *n/a* not answered, *n* number of surveys, *n/a* not answered, *SBP* spontaneous bacterial peritonitis, *BW* body weight, *AKI* acute kidney injury, *HRS* hepatorenal syndrome

The responses given to each individual question are summarized in Fig. 3a, b and Fig. 4a, b.

### Screening for varices, primary prophylaxis and secondary prophylaxis of AVB (Fig. 3a: questions 1–4 and question 7; Fig. 3c, d)

In compensated patients without varices and ongoing liver injury, only 7.1% (*n* = 5) recommended the next gastroscopy in 2 years (correct answer). Physicians who recommend the next gastroscopy in 1–2 years, i.e. 7.1% (*n* = 5), were regarded as borderline (see Fig. 3a: question 1).

Concerning question 2, when asked about primary prophylaxis in patients with compensated cirrhosis (Child-Pugh Class A5) and small EV without red spot signs (RSS), 57 (81.5%) physicians stated using nonselective beta blockers (NSBB), such as carvedilol or propranolol as a monotherapy; however, 13 (18.6%) would not start a treatment in this case, which was considered as borderline, since Billroth III recommends NSBB use in these patients. No participant stated to use EBL monotherapy or a combined treatment with NSBB and EBL.

In contrast, regarding question 3, 36 (51.4%) of the surveyed persons would perform combined treatment with NSBB and EBL and one physician would not use NSBB or EBL for primary prophylaxis in a patient with compensated cirrhosis (Child-Pugh Class A6) with big varices and red spots, which is clearly not supported by current guidelines (*see *Fig. 3c). Of note, only 33 (47.1%) physicians would treat these patients correctly with NSBB or EBL monotherapy.

In patients on NSBB without a bleeding event in the past, 40 (57.1%) of the surveyed persons would perform a surveillance gastroscopy, which is not indicated according to the current guidelines (see Fig. 3a: question 4).

Combined treatment (i.e., NSBB plus EBL) was recommended for secondary prophylaxis of variceal bleeding by the majority (*n* = 61) (see Fig. 3a*,* question 7 and Fig. 3d: 87.1% vs. 11.4%, 1.4% n/a).

### Questions addressing issues with controversial/limited evidence (Fig. 3b*: *questions 8–10)

In question 8, we addressed the use of NSBB in patients after the first occurrence of grade 3 ascites and worsening of renal function. We asked how physicians would act if the value of serum creatinine increased from 1.4 mg/dL to 1.8 mg/dL (grade 1 acute kidney injury). Only 27 (38.6%) suggested to switch the NSBB therapy from carvedilol to propranolol, while 43 (61.4%) did not choose this option.

Only 40 (57.1%) physicians would consider TIPS implantation in cases of intolerance to NSBB in secondary prophylaxis, which would have been recommended by current guidelines (see question 9).

In question 10 we asked about NSBB treatment in new onset of SBP and 32 physicians (45.7%) would pause the therapy with NSBB until spontaneous bacterial peritonitis was successfully treated. A long-term discontinuation of NSBB was recommended by 12 (17.1%) physicians and 26 (37.1%) would not interrupt/stop NSBB therapy in this case.

### Early-TIPS (Fig. 3a: questions 5 and 6)

In the last two questions of *survey‑A*, we asked about the knowledge of indications for early-TIPS.

In question 5*,* 38 subjects (54.3%) reported knowing the criteria for early TIPS; however, only 14 (20%) of the physicians listed the inclusion criteria accurately. Incomplete answers (*n* = 24, 34.3%) were considered as borderline.

In question 6 we asked for contraindications for early TIPS implantation. Of the responding physicians 52 (74.3%) correctly stated that new onset of HE at the time of acute variceal bleeding is not a contraindication for early TIPS and 1 physician did not answer this question.

### Indications for albumin in patients with cirrhosis (*see* “Supplementary Material, Table S1”)

Question 2 was an open question asking for indications for albumin substitution in patients with cirrhosis. In 53 (60.2%) cases, physicians reported hepatorenal syndrome (HRS-AKI) to be an indication for albumin treatment. In 32 (36.4%) cases, the answer was hypoalbuminemia, SBP was stated 27 (30.7%) times, after paracentesis 46 (52.3%) times, hyponatremia 5 (5.7%) times, 9 subjects (10.2%) listed severe diarrhea and malabsorption, 3 (3.4%) septic shock with cirrhosis, 3 (3.4%) acute renal failure (AKI), 1 (1.1%) HE, and 1 (1.1%) after AVB: 7 (8%) physicians did not respond to this question (*n*/a).

### Use of albumin in acute kidney injury and hepatorenal syndrome (HRS-AKI) (Fig. 4a: questions 3 and 4)

In question 3, we asked how physicians would act if the value of serum creatinine in a patient would increase from 1.4 mg/dL to 1.8 mg/dL, without an improvement after the withdrawal of diuretics. Less than half of the physicians (*n* = 40, 45.5%) stated that they would administer albumin and 5 (5.7%) physicians did not answer the question (*n*/a).

In question 4, 76 (86.4%) of the surveyed persons would use albumin in a dosage with 1 g/kg body weight, max. 100 g/day, to establish the diagnosis of HRS-AKI [2] and 6.8% reported not having easy access to albumin or albumin in a sufficient dosage; however, since they would give albumin in this case, the answer was considered borderline. Only 2 (2.3%) physicians did not recommend albumin for this patient and 4 subjects (4.5%) did not answer this question.

### Use of albumin after paracentesis and in spontaneous bacterial peritonitis (Fig. 4a: questions 6A and 6D, Fig. 4a and d)

In question 6A we asked physicians which amount of 20% albumin solution they would administer after a paracentesis of 5 l of ascites (i.e., LVP). The majority (*n* = 65, 73.9%) of the physicians stated to use the correct/recommended dose of 40 g (i.e. 200 mL of 20% albumin). 8 physicians (9.1%) indicated to use a dose of 60 g (i.e. 300 mL of 20% albumin), while 10 (11.4%) physicians would use a lower (20 g, i.e. 100 mL of 20% albumin) or a higher (80 g, i.e. 400 mL of 20% albumin) than recommended dose of albumin for LVP: 5 physicians (5.7%) did not respond to this question (Fig. 4c).

Furthermore, around two thirds of the persons interviewed on question 6D would give albumin in patients with SBP (63.6% vs. 34.1%, 2.3% n/a.). Asking for the albumin dosage in patients with SBP, there was considerable disagreement. Only 14 (15.9%) physicians answered the correct dosage according to the guidelines, while 42 (47.7%) physicians answered close to the current recommendation and were classified as borderline.

### Use of albumin in patients with infections/septic shock and cirrhosis (Fig. 4b: question 5)

There exists evidence on the use of albumin in patients with cirrhosis and non-SBP infections or septic shock. In question 5*, *20.5% (*n* = 18) would administer albumin in patients with non-SBP infections/septic shock and cirrhosis. Administering albumin in case of hypoalbuminemia was mentioned by 31 physicians (35.2%), while 37 (42%) physicians would administer crystalloid solutions or other non-albumin-based colloids.

### Special case: use of albumin in newly developed hepatic encephalopathy (Fig. 4b: question 6B)

In question 6B (see “Supplement S2 *survey‑B*”), 26 (29.5%) of the 88 interviewed persons would administer albumin after AVB with newly developed HE.

### Special case: Use of albumin in patients with cirrhosis and hyponatremia (Fig. 4b: question 6C)

The participants’ opinions regarding the use of albumin in patients with hyponatremia were divergent in question 6C. A large proportion of physicians answered not to use albumin in this situation (47.7%, *n* = 42 vs. 48.9%, *n* = 43, 3.4% n/a).

## Discussion

PH is a leading cause of hospitalization due to complications, such as variceal bleeding and ascites and causes significant morbidity and mortality in patients with cirrhosis which is also evident for Austria [14, 21, 33–36]. Therefore, an adequate prophylaxis and therapy of PH-related complication is crucial. National and international guidelines provide evidence-based recommendations regarding the management of PH [2, 13]; however, there are limited data on the actual knowledge on these recommendations and adherence to current guidelines for PH in daily clinical practice. The results of this survey indicate that many PH guideline recommendations are widely known and followed, while some recommendations are either not known or not followed in real-life [4, 5, 37, 38].

There is no strong recommendation for the use of NSBBs for primary prophylaxis in patients with small varices, since there is only a low bleeding risk [35, 39, 40]; however, a meta-analysis indicated that NSBB treatment for small varices may slow down the progression to large varices [41]. The recent PREDESCI study suggested that patients with small varices indeed benefit from NSBB therapy due to a significant risk reduction for hepatic decompensation and also for mortality [42]. In our survey, most physicians (81.5%) would start NSBB for primary prophylaxis in patients with small varices, which may reflect knowledge on the Austrian recommendations to use NSBB even for small varices. Notably, several studies have demonstrated NSBB-related benefits that are likely mediated by their additional nonhemodynamic effects [14, 18, 43].

Interestingly, many physicians (51.4%) preferred a combined treatment with NSBB plus EBL for primary bleeding prophylaxis in patients with large varices. This approach, however, represents an overtreatment, which increases the risk for severe adverse events, such as EBL-related ulcer bleeding, without being associated with a clear benefit [1, 14, 44–46]. According to current guidelines, in primary prophylaxis either nonselective beta blockers (NSBBs) or endoscopic band ligation is recommended, especially in medium to large varices [2, 13, 23].

The vast majority (81.5%) of the responding physicians in our survey would prefer carvedilol over propranolol for primary prophylaxis, which is likely related to the knowledge on the Austrian carvedilol studies showing superior reductions in portal pressure with carvedilol [36, 47]. Concerning secondary prophylaxis of variceal bleeding, the majority (87.1%) used guideline-conform combination treatment with NSBB plus EBL. In a similar Canadian survey 70.9% of physicians stated to use NSBB plus EBL for secondary prophylaxis [38].

If no varices were found in patients with compensated cirrhosis, many physicians (30%) would recommend a follow-up gastroscopy already after 1 year, which is not supported by current guidelines. The recommended endoscopic screening interval of 2 years was only followed by 7.1%, while another 7.1% stated to perform the next gastroscopy in 1–2 years. This overtreatment by short endoscopic screening intervals causes unnecessary costs and risks for the patients. Importantly, transient elastography may be used as a valuable noninvasive prescreening tool for esophageal varices when combined with a concomitant determination of the platelet count [48, 49]; however, gastroscopy is still considered important in patients with PH, especially in patients who never had gastroscopy before [2], since additional findings such as portal hypertensive gastropathy may be detected as a relevant cause of upper gastrointestinal bleeding and anemia [50–52]. Furthermore, longer screening intervals could be considered in patients with cured hepatitis C, as sustained viral response results in a profound decrease of portal pressure [53, 54].

More than a half of the responding physicians (57.1%) would perform follow-up gastroscopy in patients on primary prophylaxis with good tolerance and adequate hepatic venous pressure gradient (HVPG)-response to NSBB, which again would represent an unnecessary overtreatment not aligned with current guidelines [1, 2]. Increased knowledge of and adherence to the PH management recommendations could thus spare resources and reduce costs.

One third of physicians (38.6%) would switch bleeding prophylaxis from carvedilol to propranolol in patients with new onset ascites and rising serum creatinine values. Due to additional vasodilating effects of carvedilol, the specific safety of carvedilol (but also of traditional NSBB) in decompensated cirrhosis is still controversially debated [21, 35, 64–68]. Thus, this approach seems pathophysiologically reasonable; however, there is still limited evidence supporting this switching strategy from carvedilol to propranolol in patients with ascites and/or renal impairment [35]. Still, the Austrian [2] and European (EASL) [3] recommendations state that carvedilol should not be used in patient with severe ascites. In a similar survey, which was conducted in Denmark, 33% of the physicians stated not to stop NSBB in patients with renal impairment and 36% did not consider NSBB therapy a contraindication in patients with refractory ascites [37].

Evidence regarding the use of NSBB in patients with SBP or acute kidney injury is conflicting [35, 39, 55–57]. In our cohort, 37.1% of physicians would not stop NSBB therapy in patients with SBP. In a similar Danish survey approximately 50% of clinicians would not stop NSBB in patients with SBP [37]. Since recent data suggest that continuing NSBB during SBP is not associated with increased mortality as long as there is no severe arterial hypotension [58], the reported perceptions on how to use NSBB therapy during SBP seems to be mostly in line with current recommendations.

Knowledge on the indication criteria for early-TIPS was reported in 54.3%, but only 20% of the respondents could report these criteria correctly. This is alarming, since several trials [17, 59, 60] including an Austrian study [59, 61] have demonstrated that early-TIPS decreases not only the risk of rebleeding but also mortality in high-risk patients with variceal bleeding with a number needed to treat of only 4.

Furthermore, a lack of systematic use of early-TIPS in patients with refractory variceal bleeding requiring self-expandable esophageal metal stent implantation was also evident from another Austrian study [62]. A French survey indicated similar results with only 7% of eligible patients actually receiving early-TIPS [16]. Finally, a European multicenter study found a considerable underutilization of early-TIPS, although its use was linked to a survival benefit [63].

Most responses indicated unrestricted access to the use of albumin in patients with cirrhosis in Austria. While the established indications for albumin for LVP, SBP and HRS were almost universally correctly indicated, the reported dosing of albumin was often not adherent to the recommendations. Specifically, while 63% of physicians would administer albumin in patients with SBP, only 15.9% indicated to use the recommended dose in SBP. In a large French study, 94% of the physicians used albumin for treatment of SBP, but only 56.2% used the recommended doses of albumin [69]. In contrast, the majority of specialists (73.9%) use the correct dose of albumin in patients undergoing LVP with 8 g per liter of ascitic fluid removed [1, 2].

We also included questions regarding the use of albumin in non-established indications in the setting of cirrhosis, and about one third of physicians indicated to administer albumin in AVB and for hepatic encephalopathy. As many as 47.7% of physicians would use albumin for treatment of hyponatremia in cirrhosis, but only 20.5% would administer albumin to cirrhotic patients with non-SBP infections/septic shock. These responses are relevant, since recent studies have suggested beneficial albumin-related immunomodulatory and anti-inflammatory effects, detoxification functions, and amelioration of endothelial dysfunction in patients with cirrhosis [26, 70–74].

The spontaneous completion of the survey did not allow physicians to look up the correct answers to the questions and thus, represents a major strength of this study. Since this spontaneous survey most likely reflects true clinical practice in Austria, the actual level of knowledge on PH management and the use of albumin and the extent recommendations are followed in real-life can be sufficiently estimated. Unfortunately, we did not record how many physicians refused to answer the survey; however, the survey was well-perceived and answered by almost all approached physicians. Furthermore, to overcome the potential bias related to only asking physicians attending meetings and training courses who may be better trained than the average gastroenterologist and hepatologist, we also directly visited hospitals to ask residents, fellows, and specialists for internal medicine or gastroenterology and hepatology to fill out the questionnaires.

Although there is generally a good knowledge on the management of portal hypertension and use of albumin, there are also areas in which reported practice deviated from the national evidence-based recommendations. We think that non-adherence to recommendations should be detected in every institution that regularly treats patients with liver cirrhosis (as would be highly feasible by short questionnaires as used in this study). This would potentially allow exploring the reasons for nonadherence to the recommendations for PH management and for the use and dosing of albumin in more detail. Subsequently, specific education on particular aspects of portal hypertension can be organized in order to refresh the guideline knowledge and optimize patient management.

In conclusion, the reported management of PH and varices is mostly adherent to guidelines, but endoscopic surveillance in patients without varices is too intense and EBL is overused in the setting of PP. Knowledge on the correct use of early-TIPS must be improved among Austrian specialists. Albumin use is widely unrestricted in Austria; however, albumin is often underdosed in established indications.

## Caption Electronic Supplementary Material

Original surveys in German and their translation to English language; Supplementary Table-S1: Responses to the questions on the use of albumin in different clinical scenarios occurring in patients with cirrhosis; Supplementary Table-S2.: Details on the regions in Austria by the respondents
