# Supplementary material for: Perceptions on the management of varices and on the use of albumin in patients with cirrhosis among GI specialists in Austria
Source: Wien Klin Wochenschr. 2020 Dec 3;133(9):421–31. doi: 10.1007/s00508-020-01769-9 (PMC8116244; doi:10.1007/s00508-020-01769-9)
Supplement: Supplementary file 1 — Original surveys in German and their translation to English language; Supplementary Table-S1: Responses to the questions on the use of albumin in different clinical scenarios occurring in patients with cirrhosis; Supplementary Table-S2.: Details on the regions in Austria by the respondents [file 508_2020_1769_MOESM1_ESM.docx]

**SUPPLEMENTARY MATERIAL**

**Perceptions on the management of varices and on the use of albumin in patients with cirrhosis among GI specialists in Austria**

**TABLE OF CONTENT**

1. **SURVEY-A (German)……………………….………………………………...……2**
2. **SURVEY-A (English translation)..……………………………………………….6**
3. **SURVEY-B (German)……………………………………………....……..………10**
4. **SURVEY-B (English translation)…..…………………………………………...13**
5. **SUPPLEMENTARY TABLE……………..……………………………………….16**

Table-S1.: Responses to the questions on the use of albumin in different clinical scenarios occurring in patients with cirrhosis.

Table-S2.: Details on the regions in Austria by the respondents.

1. **SURVEY-A (German)**

Survey-A (in German) including 10 questions, which were handed out to physicians. We asked about important aspects regarding management of varices and acute variceal bleeding in their perceived daily clinical practice.

**Fragebogen Prophylaxe und Therapie von Varizenblutungen bei Patienten mit Leberzirrhose**

**Versorgen Sie Patienten mit Varizenblutungen in Ihrem Zentrum/Akut-Endoskopie?**

- Ja
- Nein

**Screening:**
1) Ein kompensierter Patient zeigt in der aktuellen Endoskopie KEINE Varizen – wann würden Sie die nächste Screening-Gastroskopie empfehlen?

**Primärprophylaxe:**

2) Patient mit kompensierter Zirrhose (Child-A5) und kleinen Varizen. Wie würden Sie die Primärprophylaxe durchführen?

a) nicht notwendig

b) Propranolol

c) Carvedilol

d) Nicht-selektive Betablocker und Ligatur

e) Ligatur

3) Patient mit kompensierter Zirrhose (Child-A6) mit großen Varizen und red spots. Wie würden Sie die Primärprophylaxe durchführen?

a) nicht notwendig

b) Propranolol

c) Carvedilol

d) Nicht-selektive Betablocker und Ligatur

e) Ligatur

4) Würden Sie Patienten, die einen nicht-selektiven Betablocker gut tolerieren (und/oder hämodynamisch anspricht=HVPG-Response) in Abwesenheit von GI-Blutungszeichen weiteren Surveillance-Gastroskopien zuführen?

- Ja
- Nein

**Varizenblutung:**

5) Kennen Sie die Einschlusskriterien der early-TIPS Indikation?

- Ja
- Nein

Wenn „Ja“ bitte zählen Sie die Kriterien auf

6) Würden Sie eine im Rahmen der Varizenblutung aufgetretene hepatische Enzephalopathie als Kontraindikation für eine early-TIPS Implantation betrachten?

- Ja
- Nein

**Sekundärprophylaxe:**

7) Patienten mit Ösophagusvarizenblutung(en) in der Anamnese sollen entweder nur endoskopische Bandligaturen oder nur nicht-selektive Betablocker erhalten. Ist das korrekt?

- Ja
- Nein

8) Eine Patientin mit großen Ösophagusvarizen erhält zur Sekundärprophylaxe (St.p. Ösophagusvarizenblutung vor ca. 8 Monaten) eine endoskopische Ligatur und Carvedilol 12.5mg 1x tgl. und wird nun wegen dem Erstauftreten von Aszites Grad 3 auf Ihrer Station aufgenommen. Im Labor zeigt sich ein geringer Anstieg im Serumkreatinin von 1.4mg/dL auf 1.8mg/dL. Es wurde die Therapie von Carvedilol 12,5mg 1x tgl. auf Propranolol 20mg (infolge max. 2x40mg pro Tag) umgestellt. Würden Sie diese Vorgangsweise unterstützen?

- Ja
- Nein

9) Würden Sie beieinem Patienten in der Sekundärprophylaxe, der eine Intoleranz auf NSBBs entwickelt und schweren, jedoch nicht-refraktären Aszites hat, die Indikation zur TIPS-Implantation stellen?

- Ja
- Nein

10) Würden Sie bei Patienten mit Ösophagusvarizen und -blutung in der Anamnese bei normotensiven Blutdruckwerten m RR und normalem Serum-Kreatinin beim Auftreten einer SBP die Gabe von NSBB beenden?

- Ja – beenden für immer
- Ja, aber nur pausieren bis SBP ausheilt
- Nein

**2. SURVEY-A (English translation)**

Survey-A (in English) including 10 questions, which were handed out to physicians. We asked about important aspects regarding management of varices and acute varicea bleeding in their perceived daily clinical practice.

**Survey on management of varices and variceal bleeding in patients with liver cirrhosis**

**Do you treat patients with acute variceal bleeding at your department/ institution?**

- Yes
- No

**Screening:**
1) A compensated patient shows NO esophageal varices at upper GI endoscopy – would you recommend to perform the next screening endoscopy for this patient?

**Primary prophylaxis:**

2) A patient with compensated cirrhosis (Child-A5) shows small esophageal varices. How would you perform primary bleeding prophylaxis in this patient?

a) I would not yet recommend any type of primary prophylaxis in this patient

b) Propranolol only

c) Carvedilol only

d) non-selective betablockers plus endoscopic band ligation

e) endoscopic band ligation only

3) A patient with compensated cirrhosis (Child-A6), large varices and red spot signs. How would you perform primary bleeding prophylaxis in this patient?

a) I would not yet recommend any type of primary prophylaxis in this patient

b) Propranolol only

c) Carvedilol only

d) non-selective betablockers plus endoscopic band ligation

e) endoscopic band ligation only

4) Would you perform further surveillance-gastroscopies in patients taking non-selective betablocker with good/adequate hemodynamic (HVPG-Response) response and no signs of GI bleeding ?

- Yes
- No

**Acute Variceal Bleeding:**

5) Do you know the inclusion criteria for early-TIPS?

- Yes
- No

If „Yes“, please list all criteria of early-TIPS

6) Do you consider a new onset of hepatic encephalopathy developing at the time of acute variceal bleeding as a contraindication for early-TIPS implantation?

- Yes
- No

**Secondary prophylaxis:**

7) Patients with a history of acute variceal bleeding should either be only treated with endoscopic band ligation or only with non-selective betablockers. Is that correct?

- Yes
- No

8) A female patient with large esophageal varices receives secondary prophylaxis (previous history of variceal bleeding about 8 months ago) with endoscopic band ligation plus non-selective betablockers. Now, she is admitted to your ward because of new onset of ascites grade 3. In the laboratory results there is an increase of serum creatinine from 1.4mg/dL to 1.8mg/dL. The therapy was switched from carvedilol 12.5mg once daily to propranolol 20mg (slowly titrated to a maximum of 2x40mg daily). Would you agree with this treatment approach?

- Yes
- No

9) Would you consider a TIPS implantation in patients with secondary prophylaxis and severe, but non-refractory ascites, who is intolerant (or develops intolerance) to non-selective betablockers?

- Yes
- No

10) Would you stop therapy with non-selective betablockers in a patients with previous variceal bleeding, who now develops spontaneous bacterial peritonitis (SBP) but has normal blood pressure and normal serum creatinine.

- Yes – discontinue betablocker and never restart again
- Yes, pause betablockers until SBP is resolved.
- No

**3. SURVEY-B (German)**

Survey-B (in German) including 9 questions, which were handed out to physicians. We asked about important aspects regarding their restricted versus unrestricted access to albumin, and the use and dosing of albumin in their perceived daily clinical practice.

**Fragebogen zur Gabe von Humanalbumin bei Patienten mit portaler Hypertension**

1. Haben Sie uneingeschränkten Zugang zu Humanalbumin in Ihrem Zentrum?

- Ja
- Nein

Wenn „Nein“, geben Sie bitte an welche Schritte Sie unternehmen müssen bevor Sie Humanalbumin verwenden können.

2) Bei welchen Indikationen verwenden Sie in Ihrem Zentrum Humanalbumin? Bitte zählen Sie diese auf:

3) Im Labor zeigt sich ein geringer Anstieg im Serumkreatininwert von 1.4mg/dL auf 1.8mg/dL. Dieser Wert ist auch nach Pausieren der Diuretika nicht rückläufig. Ist die Gabe von Humanalbumin in Ihren Augen indiziert?

- Ja
- Nein

4) 1g/kg KG Humanalbumin (d.h. zB. 2x200mL 20% Humanalbumin bei 80kg täglich) in Kombination mit Terlipressin ist Standardtherapie eines HRS-1 (HRS-AKI). Würden Sie (und haben Sie die Möglichkeit) das in Ihrem Zentrum so zu dosieren?

- Ja, ich würde – kann aber nicht
- Ja, ich würde – kann es auch so geben
- Nein, würde ich nicht, glaube nicht an die Effektivität

5) Setzen Sie Humanalbumin als Mittel der Wahl zur Volumengabe bei Infekionen/Septischen Schock bei Patienten mit Leberzirrhose ein?

- Ja, immer
- Ja, aber nur wenn Serum-Albumin erniedrigt
- Nein, ich gebe primär Kristalloide
- Nein, ich gebe primär andere Kolloide

6) **Fallbeispiele:**

1. Bei einer Patientin wurden 5l Aszites abpunktiert. Wie viel 20% Humanalbumin werden dieser Patientin in Ihrem Zentrum verabreicht?
2. 100mL
3. 200mL
4. 300mL
5. 400mL
6. Ein Patient entwickelt kurz nach einer akuten Ösophagus- Varizenblutung eine hepatische Enzephalopthie. Ist Substitution von Humanalbumin in Ihren Augen indiziert?

- Ja
- Nein

1. Ein Patient mit Hyponatriämie hat auch einen erniedrigten Serum-Albumin-Wert von 27g/dL. Würden Sie ihm Humanalbumin verabreichen wollen? Wenn ja, haben Sie in Ihrem Zentrum die Möglichkeit?

- Ja
- Nein

1. Ein Patient mit Child-Pugh B entwickelt eine spontan bakterielle Peritonitis. Ist Substitution von Humanalbumin in Ihren Augen indiziert?

- Ja
- Nein

Wenn ja, wieviel (Dosisangabe %HA/Volumen) verabreichen Sie in Ihrem Zentrum?

**4. SURVEY-B (English translation)**

Survey-B (in English) including 9 questions, which were handed out to physicians. We asked about important aspects regarding their restricted versus unrestricted access to albumin, and the use and dosing of albumin in their perceived daily clinical practice.

**Survey on the use of albumin in patients with portal hypertension**

1. **Do you have** unrestricted access to albumin at your department/institution?

- Yes
- No

If „no“, please state which steps are necessary before you are able to use albumin

2) Please provide a list of indications for which you are able to use albumin at your institution/hospital.

3) A patient shows an increase of serum creatinine from 1.4mg/dL to 1.8mg/dL and no improvement is observed after discontinuation of the diuretics. Would you use albumin in this case?

- Yes
- No

4) A combination of 1g/kg body weight albumin (2x200mL 20% albumin for 80kg body weight daily) and Terlipressin is the standard treatment of hepatorenal syndrome (HRS-AKI). Would you agree with this dosing of albumin and are you able to use these doses of albumin at your instituation/center?

- Yes, i would, but I do not have the option to use this dose of albumin
- Yes, i would and I can usethis dose of albumin
- No, i would not use albumin because I do not think that albumin is effective in this setting

5) Do you use albumin as the first choice for resuscitation in patients with liver cirrhosis developing infections or septic shock

- Yes, always
- Yes, but only if the serum albumin levels are low
- No, i prefer crystalloid solutions
- No, i prefer other non-albumin colloids

6) **Case examples:**

1. 5L of ascitic fluid were drained from a patient with liver cirrhosis. How much volume of 20% albumin would you recommend to administer?
2. 100mL
3. 200mL
4. 300mL
5. 400mL
6. A patient developed hepatic encephalopathy shortly after acute variceal bleeding. Would you consider to use albumin in this setting?

- Yes
- No

1. A patient with hyponatremia has also a low level of albumin (27g/dL). Would you recommend to administer albumin in this patient?

- Yes
- No

1. A patient with Child-Pugh B develops spontaneous bacterial peritonitis. Is the administration of albumin indicated in this patient?

- Yes
- No

If yes, how much (dosage information %HA per volume) would you administer?

**5. SUPPLEMENTARY TABLE**

**Table-S1. Responses to the questions on the use of albumin in different clinical scenarios occurring in patients with cirrhosis.**

|  | n=88 (%) |
| --- | --- |
| HRS-AKI | 53 (60.2) |
| Hypalbuminemia | 32 (36.4) |
| SBP | 27 (30.7) |
| Paracentesis | 46 (52.3) |
| Hyponatremia | 5 (5.7) |
| Diarrhea and malabsorption | 9 (10.2) |
| Septic shock | 3 (3.4) |
| AKI | 3 (3.4) |
| HE | 1 (1.1) |
| AVB | 1 (1.1) |
| n/a | 7 (8) |

Abbreviations: HRS-AKI= Hepatorenal syndrome, SBP= spontaneous bacterial peritonitis, AKI=Acute kidney injury, HE= hepatic encephalopathy, AVB= acute variceal bleeding, n= number of patients, n/a= not answered.

**Table-S2.: Details on the regions in Austria by the respondents.**

| **Completed questionnaires** | n=158 (%) |
| --- | --- |
| Only information on state (excl. Vienna) | 40 (25.3%) |
| Larger cities | 91 (57.6%) |
| Smaller cities | 27 (17.9%) |
| Survey-A n=70 (%) | |
| Only information on state (excl. Vienna) | 16 (22.9%) |
| Larger cities | 44 (62.9%) |
| Smaller cities | 10 (14.3%) |
| Survey-B n=88 (%) | |
| Only information on state (excl. Vienna) | 24 (27.3%) |
| Larger cities | 47 (53.4%) |
| Smaller cities | 17 (19.3%) |

Abbreviations: n=number of patients, excl.=excluded
